# The Sorting and Transport of the Cargo Protein CcSnc1 by the Retromer Complex Regulate the Growth, Development, and Pathogenicity of *Corynespora cassiicola*

**DOI:** 10.3390/jof10100714

**Published:** 2024-10-14

**Authors:** Shuyuan Cheng, Yunfei Long, Xiaoyang Zhang, Bing Liu, Shuilin Song, Genghua Li, Yuzhuan Hu, Lei Du, Quanxing Wang, Junxi Jiang, Guihong Xiong

**Affiliations:** 1 College of Agronomy, Jiangxi Agricultural University, Nanchang 330045, China; chengshuyuan2020@163.com (S.C.); lbzjm0418@126.com (B.L.); songshuilin2024@126.com (S.S.); 13807914680@163.com (G.L.); 13640111730@163.com (Y.H.); touleibb@gmail.com (L.D.); 15193502750@163.com (Q.W.); 2 Jiangxi Province Key Laboratory of Vegetable Cultivation and Utilization, Jiangxi Agricultural University, Nanchang 330045, China; 3 College of Plant Protection, Fujian Agriculture and Forestry University, Fuzhou 350002, China; longyunfei2023@163.com; 4 Jiujiang Agricultural Technology Extension Center, Jiujiang 332000, China; zxygo123@163.com

**Keywords:** retromer, cargo sorting, fungal development, pathogenicity, *Corynespora cassiicola*

## Abstract

In eukaryotes, the retromer complex is critical for the transport of cargo proteins from endosomes to the trans-Golgi network (TGN). Despite its importance, there is a lack of research on the retromer-mediated transport of cargo proteins regulating the growth, development, and pathogenicity of filamentous fungi. In the present study, transcriptome analysis showed that the expression levels of the retromer complex (*CcVPS35*, *CcVPS29* and *CcVPS26*) were significantly elevated during the early stages of *Corynespora cassiicola* invasion. Gene knockout and complementation analyses further highlighted the critical role of the retromer complex in *C. cassiicola* infection. Subcellular localization analysis showed that the retromer complex was mainly localized to the vacuolar membrane and partially to endosomes and the TGN. Further research found that the retromer core subunit CcVps35 can interact with the cargo protein CcSnc1. Subcellular localization showed that CcSnc1 is mainly located at the hyphal tip and partially in endosomes and the Golgi apparatus. Deletion of *CcVPS35* resulted in the missorting of CcSnc1 into the vacuolar degradation pathway, indicating that the retromer can sort CcSnc1 from endosomes and transport it to the TGN. Additionally, gene knockout and complementation analyses demonstrated that *CcSnc1* is critical for the growth, development, and pathogenicity of *C. cassiicola*. In summary, the vesicular transport pathway involving the retromer complex regulates the sorting and transport of the cargo protein CcSnc1, which is important for the growth, development and pathogenicity of *C. cassiicola.*

## 1. Introduction

Kiwifruit is widely acknowledged as the “king of fruits” due to its exceptionally high concentration of vitamins, minerals, and other nutrients [1]. The kiwifruit industry plays a vital role in the regional economy of China, serving as a key driver of rural revitalization. Corynespora leaf fall (CLF) is a major threat to kiwifruit cultivation in China, causing severe yield losses, primarily due to the fungal pathogen *Corynespora cassiicola* [2]. Moreover, *C. cassiicola*, a necrotrophic parasitic fungus, poses a widespread threat to plant health, inducing symptoms in leaves, stems, roots, flowers, and fruits, affecting over 500 plant species [3]. Therefore, a comprehensive understanding of the mechanisms involving growth and pathogenicity will facilitate the development of efficient management strategies against *C. cassiicola*.

Conidia, the main infection type of fungal pathogens in the field, germinate on the plant surface and then form germ tubes, appressoria and other structures, promoting the invasion of plant tissues [4,5]. Hyphal extension is essential for *C. cassiicola* colonization and aggressiveness, the related gene products of which are involved with biochemical functions [6,7], such as cutinases, cell wall degrading enzymes, and the toxin cassiicolin [8,9]. So far, only a few pathogenicity-related genes, including *CCk1*, *CMP1*, and *Cas*, have been cloned and characterized [10,11,12]. However, many pathogenicity-related genes still need to be identified and characterized.

In eukaryotic cells, vesicle transport plays a crucial role in coordinating numerous cellular functions [13]. This process involves the exchange of macromolecules like proteins and lipids between subcellular organelles [14]. It is therefore essential to analyze the action of proteins involved in vesicular transport, such as the retromer complex, which enables retrograde transport of membrane proteins from endosomes to the trans-Golgi network (TGN) or plasma membrane [15]. Previous studies have highlighted the significant impact of the retromer complex on the development and virulence of *Fusarium graminearum and Magnaporthe Oryzae* [16,17,18]. The retromer complex is a conserved and essential component of the endosomal protein-sorting machinery, related to the cytoplasmic endosomal surface, mediating the retrograde transport of transmembrane cargo from endosomes to the Golgi apparatus [19]. It consists of two subcomplexes: the vacuolar protein sorting trimers Vps35, Vps29, and Vps26, known as the cargo selection complex (CSC), along with the sorting nexin (SNX) protein dimers Vps5 and Vps17 [20]. Loss or dysfunction of the retromer, along with protein mislocalization, can lead to various pathological conditions [21]. As the core protein of the retromer complex, Vps35 directly interacts with cargo proteins due to its cargo specificity. The role of Vps35 and the retromer has been confirmed to regulate plant infection in fungal pathogen [18]. To the best of our knowledge, the functions of Vps35 and the retromer have not been verified in regulating kiwifruit infection by *C. cassiicola.* Simultaneously, we found a protein named Snc1 that interacts with Vps35. Snc1 is a soluble N-ethylmaleimide-sensitive factor attachment receptor (SNARE) protein, which is considered to be a type of retromer-regulated v-SNARE [22]. Recent research shows that the retromer facilitates the intracellular sorting of the v-SNARE MoSnc1 from the vacuole to the cell surface, thereby contributing to effector secretion [16]. Consequently, we also plan to explore the role of Snc1 in *C. cassiicola*.

In this study, we delved into the relative transcription levels of genes during *C. cassiicola* infection in kiwifruit leaves using RNA-Seq. A total of 92.75 GB of raw data were obtained via transcriptome sequencing. Functional annotations of differentially expressed genes (DEGs) were performed using Gene Ontology (GO), Swiss-Prot, and the Kyoto Encyclopedia of Genes and Genomes (KEGG). The genes of proteins from the different pathways showed significant differences at various stages of infection, particularly those involved in vesicle transport. Remarkably, the expression levels of the retromer complex subunit genes *CcVPS35*, *CcVPS29* and *CcVPS26* significantly escalated during infection by *C. cassiicola*. Due to the loss of the retromer complex, a pronounced impairment was observed in the growth and virulence of *C. cassiicola*. The retromer complex in *C. cassiicola* predominantly localized to the vacuole membrane, with partial localization in the endosome and late Golgi apparatus. Additionally, CcSnc1 was identified as a cargo protein of the retromer-mediated retrograde mechanism. Disruption of the retromer transport mechanism resulted in mislocalization of the CcSnc1 cargo to the vacuolar degradation pathway.

Furthermore, the loss of *CcSNC1* significantly impacted the growth and development of *C. cassiicola*, which was similar to the loss of the retromer complex. In summary, our findings indicated that the retromer complex mediated the transport of the cargo protein CcSnc1, thus regulating the growth, development, and pathogenicity of *C. cassiicola*.

## 2. Materials and Methods

### 2.1. Fungal Strains and Growth Conditions

The *C. cassiicola* strain SK-4 was utilized as the wild type in this study. All the mutants were derived from SK-4, as listed in Table S1. The SK-4 and mutant strains were inoculated on Potato Dextrose Agar medium (PDA: 200 g potato, 20 g glucose, 15 g agar) at 28 °C for 7 days.

### 2.2. Sample Preparation

Kiwifruit leaves of the “Hongyang” cultivar were collected from the Shankou orchard in Fengxin county, Jiangxi province. Seven-week-old leaves were inoculated with 10 µL of a *C. cassiicola* conidial suspension (10^6^ spores/mL) per drop on the leaf surface using droplet inoculation and then cultured in a humid chamber at 28 °C. The leaves were rapidly frozen in liquid nitrogen and subsequently stored at −80 °C for RNA extraction after inoculation at 24 h and 72 h. The conidia suspension for inoculation at 0 h post infection (hpi) was used as a control. Each experiment was repeated three times.

### 2.3. RNA Extraction and RNA Sequencing

The total RNA was extracted from the inoculated Kiwifruit leaves using the TRIzol Total RNA Isolation Kit (Life Technologies, Shanghai, China). The RNA was purified by adsorption columns and collection pipes. The integrity and quality of the RNA were checked by an Implen NanoPhotometer^®^ (Munich, Germany) P330 ultra-micro spectrophotometer. The RNA was prepared from three independent biological replicates. The RNA library was prepared using the NEB Nextfi Ultra RNA Library Prep Kit (New England Biolabs, Ipswich, MA, USA). The quality of the cDNA library was assessed using the Agilent 2100 Bioanalyzer and ABI Step One Plus Real-Time PCR System. RNA sequencing was conducted on the DNBSEQ platform at the Beijing Genomics Institute (Beijing, China).

### 2.4. Bioinformatic Analysis

High-quality clean sequence reads from *C. cassiicola* were utilized for the bioinformatic analysis. Paired-end clean sequence reads were mapped to the reference genome of *C. cassiicola* (taxid: 1448308) using HISAT software (v2.2.0) [23]. Gene annotation was performed using public databases: KEGG, GO and Swiss-Prot. Differentially expressed genes (DEGs) were identified by comparing the levels of gene expression at 24 and 72 hpi with the control (0 hpi) [24,25]. Between-sample differential gene analysis was conducted with DEseq2 under the conditions of Fold Change ≥ 2 and FDR ≤ 0.1. A heatmap of differential gene clusters was generated using the pheatmap function. Differentially expressed genes were functionally classified based on the GO and KEGG annotation results. GO and KEGG enrichment analyses were conducted using the phyper function in R software (v4.2.0). Candidate genes with a Q value of ≤ 0.05 were considered significantly enriched [26].

### 2.5. qRT-PCR

The primers used for the RT-qPCR assays are shown in Table S2. The relative expression levels of the genes were detected by RT-qPCR using the Hieff^®^ qPCR SYBR Green Master Mix (YE SEN, Shanghai, China) in a CFX 96TM Real-Time System (Bio-Rad, Hercules, CA, USA). Actin served as the housekeeping control. The relative expression was calculated by the cycle threshold (2^−ΔΔCt^) method [27]. The experiments were replicated three times.

### 2.6. Construction of Gene Deletion Mutants and Complementation

The protoplast preparation and fungal transformation of *C. cassiicola* were conducted according to established protocols [28]. A split-marker approach was used to generate gene deletion mutants lacking retromer complex subunit genes and encoding *CcSNC1* [29]. The deletion mutants were obtained using homologous recombination, and the deletions were confirmed by PCR and qRT-PCR (Figures S1–S3). To construct the complementation strains, the coding regions and native promoters of *CcVPS35*, *CcVPS29*, *CcVPS26* and *CcSNC1* were amplified and cloned into the pKNT-GFP vector [18]. Plasmids were transformed into the *CcVPS35*, *CcVPS29*, *CcVPS26* and *CcSNC1* deletion mutants strains by PEG-mediated protoplast transformation. The primers utilized to amplify the flanking sequences for each gene are listed in Table S3. Detailed information on the plasmid construction in this study is listed in Table S4.

### 2.7. Fungal Growth and Pathogenicity Assays

For the growth assays, conidia of SK-4, deletion mutants, and complemented strains were inoculated on PDA medium. Mycelial growth was observed on PDA for 7 days following inoculation. For the pathogenicity assay, 10 μL conidia suspension (1.0 × 10^6^ conidia/mL) of the tested strains was dropped on kiwifruit leaves and incubated at 28 °C in the dark for 72 h. The development of disease was observed and the lesion area was measured every 12 h after inoculation. All the experiments were performed in triplicate.

### 2.8. Yeast Two-Hybrid (Y2H), Co-Immunoprecipitation (Co-IP) and Bimolecular Fluorescence Complementation (BiFC) Assays

The Y2H test was conducted according to established protocols [17,18]. The full-length ORFs of *CcVPS35*, *CcVPS29* and *CcVPS26* were separately introduced to the prey vector pGADT7 and the bait pGBKT7. The primers used are listed in Table S3. The bait and prey plasmids were co-transformed into the yeast AH109 cells. The transformants were cultured on Synthetic Dropout medium SD/-Trp-Leu and then positive colonies were grown on SD/-Trp-Leu-His-Ade/X-a-gal medium. The positive and negative controls in the assay were pGBKT7-53/pGADT7-T and pGBKT7-Lam/pGADT7-T, respectively.

For the Co-IP assays, vegetative hyphae were obtained by culturing mycelial plugs of the strains expressing the fusion proteins in PDB liquid media at 28 °C, 110 rpm, for 3 days. The mycelia were lysed in lysis buffer (Sangon Biotech, Shanghai, China). The total cell lysates were subsequently incubated with Anti-GFP beads (ChromoTek Inc., Munich, Germany) at 4 °C for 4 h. The bound proteins, eluted with protein-loading buffer, were heated at 100 °C for 10 min. The proteins were then separated by 10% SDS-PAGE and transferred to PVDF membranes for Western blot analysis.

For the BiFC assay, various pairs of constructs and single constructs were introduced into SK-4 protoplasts. Four types of transformants with expression of a gene encoding a fusion of the test protein with the N-terminal fragment of yellow fluorescence protein (nYFP) and/or the C-terminal fragment of this protein (cYFP), or encoding nYFP or cYFP alone, were used as negative controls, including *CcVPS35-cYFP*+*nYFP*, *cYFP*+*CcVPS26-nYFP*, *cYFP*+*CcVPS29-nYFP* and *CcVPS29-cYFP*+*nYFP*. Transformants resistant to both hygromycin and/or neomycin were isolated and confirmed by PCR [30,31]. The YFP signals were then examined using a laser scanning confocal microscope (Nikon, Tokyo, Japan).

### 2.9. Staining and Live Cell Imaging of C. cassiicola

To observe the vacuolar membrane, vegetative hyphae were stained with FM4-64 (Eugene, OR, USA) at a final concentration of 10 μg/mL for 30 min. A mycelial block containing the leading hyphae was positioned upside down on coverslips, and then observed by laser scanning confocal microscope. Images were captured within a single focal plane.

### 2.10. Phylogenetic Analysis and Protein Structure Prediction

The amino acid sequences of CcVps35, CcVps29, CcVps26, CcSnc1 and other reference strains were obtained from the NCBI database (https://www.ncbi.nlm.nih.gov/, accessed on 20 April 2024). The protein structures of CcSnc1 were predicted using A_LPHA_F_OLD2_ [32]. A neighbor-joining tree was constructed based on the amino acid sequences using MEGA 7.0, where the number at the nodes represents the percentage of their occurrence in 1000 bootstrap replicates [33,34].

## 3. Results

### 3.1. Infection of Kiwifruit Leaves by C. cassiicola

It is evident that kiwifruit leaves inoculated with *C. cassiicola* exhibited a significant decay at 72 hpi, while the controls showed no symptoms (Figure S4a). At 24 hpi, the decay diameter measured approximately 5.59% (Figure S4b). With a prolonged culture time, the decay diameter was expanded notably and reached 32.89% at 72 hpi (Figure S4c). Microscopic observations showed that the spores of *C. cassiicola* appeared cylindrical or inverted rod-shaped on onion epidermis (Figure S4d). The spores produced germ tubes (GT) at 3 hpi, which gradually elongated over time and developed into hyphae (Figure S4e). Concurrently, superficial hyphae (SH) continued to proliferate on the surface during host tissue penetration. Subsequently, the radiating hyphae (RH) differentiated, while the lesion spread (Figure S4f).

### 3.2. RNA-Seq Analysis and Validation

**Figure 1:**
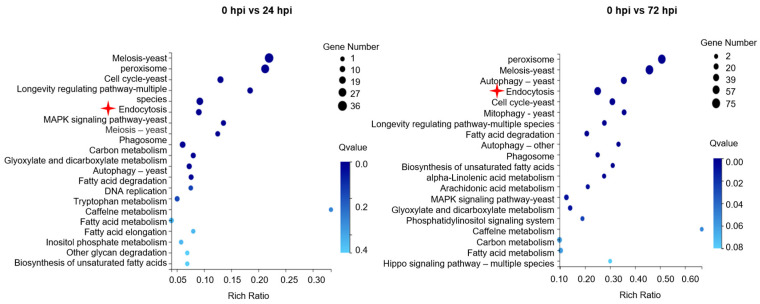
Top 20 most altered cellular processes in *C. cassiicola* during infection. Analysis revealing mRNA enrichment for proteins from 20 cellular processes during interaction in *C. cassiicola* after 24 hpi (**left**) and after 72 hpi (**right**). The rich ratio represents the ratio of the number of DEGs attributed to a particular process to the number of total genes annotated in this process. The higher the rich ratio, the greater the degree of enrichment. Notably, asterisks denote the presence of endocytosis among other cellular processes.

**Figure 2:**
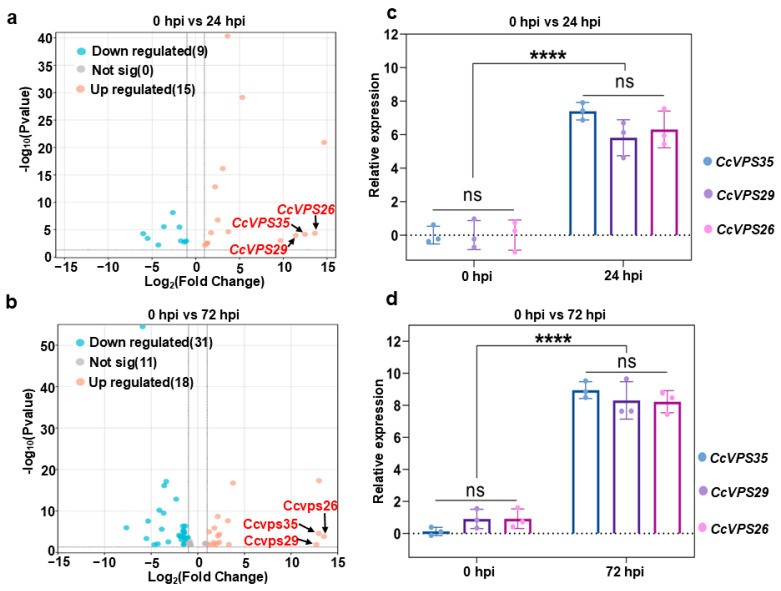
Significant differences in the expression of endocytosis-related genes demonstrated by changes in the expression levels of *CcVPS35*, *CcVPS29* and *CcVPS26*. (**a**,**b**) Significantly up- and down-regulated endocytic pathway genes in *C. cassiicola* at 24 hpi and 72 hpi identified by RNA-seq; blue dots represent down-regulated genes, pink dots represent up-regulated genes and gray dots represent genes with no significant differences in expression. The grey line represents the criteria for changes in gene expression. The black arrows indicate the gene expression levels of *CcVPS35*, *CcVPS29* and *CcVPS26*. (**c**,**d**) qRT-PCR-based quantification of the expression levels of genes encoding CcVps35, CcVps29 and CcVps26 retromer subunits at 24 hpi and 72 hpi. Box-plot values represent the means of independent experiments. Statistical analysis was processed by one-way ANOVA for multiple comparisons using GraphPad Prism 9 (**** *p* < 0.0001; ns, not significant at *p* > 0.05).

A total of 92.75 Gb of clean reads were obtained by RNA-Seq. The clean reads of *C. cassiicola* (0 hpi) were 67,771,404, 67,871,064, and 70,898,154. However, the clean reads of *C. cassiicola* (24 hpi) were 67,433,702, 68,934,824, and 70,231,596, while they were 68,789,380, 67,545,610, and 68,863,100 at 72 hpi. The Q_30_ of the clean reads exceeded 89.27%, suggesting that the transcriptome sequencing yielded a sufficient number of high-quality reads for subsequent analyses (Table S5). In addition, the DEGs were functionally annotated using GO, Swiss-Prot and KEGG bases. The 20 most enriched KEGG pathways in *C. cassiicola* are shown in Figure S5. To validate the accuracy of the transcriptomic analysis results, sixteen DEGs were randomly selected for RT-qPCR analysis. The relative expression levels obtained by qRT-PCR were consistent with those of RNA-seq (Figure S6, Table S6). Interestingly, among the top 20 DEGs of cellular processes, the expression levels of endocytosis-related genes exhibited significant differences at 24 hpi and 72 hpi (Figure 1). Specifically, among the genes encoding proteins involved in endocytosis, 15 were upregulated and 9 down-regulated at 24 hpi, while 18 were up-regulated and 31 down-regulated at 72 hpi. Notably, the expression levels of *CcVPS35*, *CcVPS29* and *CcVPS26* showed continuous up-regulation at both 24 hpi and 72 hpi (Figure 2a,b). The CcVps35, CcVps29 and CcVps26 proteins, encoded by the mentioned genes, respectively, are subunits of the retromer complex involved in the transport of vesicles from endosomes to the TGN or plasma membrane, and they are essential for the growth, development and pathogenicity of filamentous fungi [17,18,35]. To determine whether the retromer complex plays a role in *C. cassiicola* infection, qRT-PCR was performed. The expression of genes encoding the retromer subunits Vps35, Vps26 and Vps29 during *C. cassiicola* infection was quantified. The results demonstrated high expression levels of the coding genes of the retromer subunits at 24 hpi and 72 hpi (Figure 2c,d). Together, these results demonstrated that the retromer complex may play a positive role in the infection of *C. cassiicola*.

### 3.3. Retromer Components Are Conserved in C. cassiicola

Previous studies have shown that the retromer complex in yeast is composed of three vacuolar sorting proteins: Vps35, Vps29, and Vps26 [35,36]. The genes encoding the retromer subunits CcVps35 (BS50DRAFT_573581), CcVps26 (BS50DRAFT_652902), and CcVps29 (BS50DRAFT_490648) were screened by RNA-Seq analysis and confirmed by reference genome of *C. cassiicola* Philippines (GCA_003016335.1_Corynespora_cassiicola_v1.0).

Phylogenetic analysis of CcVps35, CcVps29, and CcVps26 across fungi, plants, and mammals revealed their ancient origins and distinct conservation patterns (Figures S7–S9).

**Figure 3:**
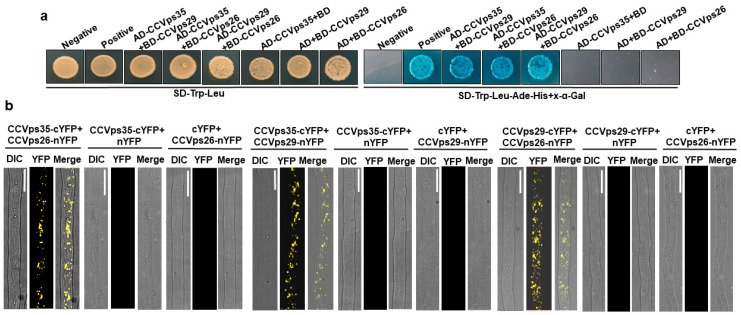
Interaction between CcVps35, CcVps29 and CcVps26. (**a**) Yeast two-hybrid (Y2H) assay showing positive interactions among the retromer components (CcVps35, CcVps29, CcVps26) in *C. cassiicola*. The positive and negative controls in the assay were pGBKT7-53/pGADT7-T and pGBKT7-Lam/pGADT7-T. The AD+BD-*CcVps35/29/26* showing the yeast cells transformed with single plasmids. (**b**) BiFC assay showing the interactions among CcVps35, CcVps29 and CcVps26 in vivo. DIC: differential interference contrast. Scale bar 10 μm.

In yeast, plants, and mammals, Vps26 and Vps29 constitute the cargo-selective subcomplex of the retromer through their interaction with Vps35 [37,38]. However, the specific interaction pattern among these retromer subunits in *C. cassiicola* remains unknown. Yeast two-hybrid (Y2H) and BiFC assays were performed to detect the interaction between the retromer subunits. We found that CcVps35 strongly interacted with CcVps26 and CcVps29 in yeast cells (Figure 3a). The BiFC assay further confirmed these interactions (Figure 3b). Therefore, our results revealed that CcVps35, CcVps26 and CcVps29 interacted with each other in *C. cassiicola*.

### 3.4. The Retromer Complex Is Essential for Hyphal Growth and Pathogenicity

**Figure 4:**
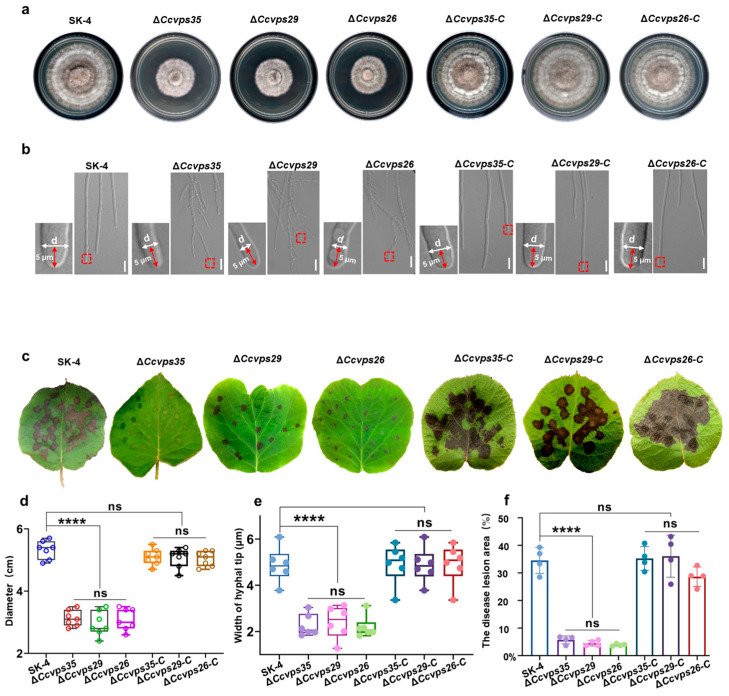
*CcVps35*, *CcVps29* and *CcVps26* are involved in the growth and pathogenicity of *C. cassiicola*. (**a**) Colonies of the wild type (SK-4), each gene deletion mutant (Δ*Ccvps35*, Δ*Ccvps29* and Δ*Ccvps26*), and their respective complemented strains (Δ*Ccvps35-C*, Δ*Ccvps29-C* and Δ*Ccvps26-C*) grown on PDA for 7 d. (**b**) Hyphal tip growth and branching patterns of the tested strains on PDA. Δ*Ccvps35*, Δ*Ccvps29* and Δ*Ccvps26* showed significant defects in polarized growth and cell expansion at the hyphal tip. The red arrow represents 5μm from the hypha tip. The red dotted box represents the measurement position. “d” represents the diameter of hyphae at 5 μm from the tip of the hyphae. (**c**) Symptoms on kiwifruit leaves inoculated with conidia from the tested strains at 72 hpi. (**d**) Colony growth rate test of the mutants, complemented strains and SK-4 strain. (**e**) Statistical analysis of the diameter differences of 5 μm width from the hyphal tip between the mutants, complemented strains and SK-4 strains. (**f**) Pathogenicity assay for the mutants, complemented strains and SK-4 strains. The disease lesion area (%) indicated the percentage of lesion size area to total leaf area. The values shown are the means of independent experiments. Statistical analysis was processed by one-way ANOVA for multiple comparisons using GraphPad Prism 9 (**** *p* < 0.0001, ns, not significant at *p* > 0.05). DIC: differential interference contrast. Scale bar 10 μm.

To explore the biological functions of the retromer complex in *C. cassiicola*, the wild-type strain SK-4, deletion mutants (Δ*Ccvps35*, *ΔCcvps29* and Δ*Ccvps26*), and complemented strains (Δ*Ccvps35-C*, Δ*Ccvps29-C* and Δ*Ccvps26-C*) were cultured on PDA at 28 °C for 7 days to compare their growth rates. The experiment showed that the growth rate of the retromer complex mutant was significantly reduced compared to that of SK-4 and the complemented strains (Figure 4a,d). Furthermore, the deletion mutants had abnormal morphology in the hyphae. The microscopic observations revealed that the hyphae of the mutant strains failed to maintain stable polarized growth, exhibiting curved growth compared to the straight growth observed in the SK-4 and complemented strains (Figure 4b). Measurement of the width of the hyphae 5 µm from the tip illustrated a significant reduction in the diameter compared to the SK-4 and complemented strains (Figure 4e). Consequently, the results indicate that the retromer complex is essential for the normal vegetative growth of *C. cassiicola*.

We then further tested the pathogenicity of the deletion mutant. Kiwifruit leaves were inoculated with conidia suspension of the SK-4, mutant, and complemented strains, respectively. Pathogenicity detection revealed that the SK-4 and complemented strains induced numerous typical brown spots on the leaves, while the mutant only produced small lesions at 72 hpi (Figure 4c). The disease lesion area retromer mutants had a significant reduction compared to the SK-4 and complemented strains (Figure 4f). Overall, the results suggest that the retromer complex is essential for vegetative growth and pathogenicity in *C. cassiicola*.

### 3.5. Retromer Complex Predominantly Localizes to the Vacuole Membrane

**Figure 5:**
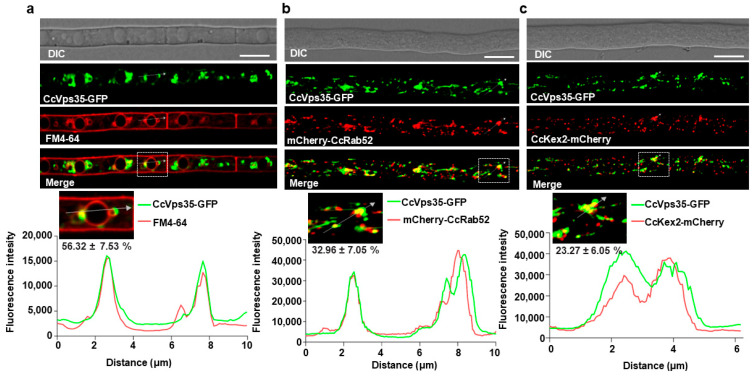
Subcellular localization of CcVps35 in *C. cassiicola*. (**a**) Hyphae expressing CcVps35-GFP were stained with FM4-64 and observed under a confocal microscope. CcVps35-GFP is localized to the vacuolar membrane and partially to endosomes and the TGN, which co-localized with the endocytic dye FM4-64, and the co-localization rate is 56.32 ± 7.53%. (**b**) CcVps35-GFP co-localized with the endosomes marker mCherry-CcRab52, and the co-localization rate is 32.96 ± 7.05%. (**c**) CcVps35-GFP co-localizes with the TGN marker CcKex2-mCherry, and the co-localization rate is 23.27 ± 6.05%. White arrows indicate the co-localization sites. DIC: differential interference contrast. The white dotted box represents the measurement position. Scale bar 10 μm.

Previous studies have established that the retromer complex primarily localizes to the vacuole [17,37]. We checked the localization of CcVps35. The results revealed that the retromer core subunit CcVps35 localized to cytoplasm with punctates in the vegetative hyphae (Figure S10). To further investigate the subcellular localization of CcVps35 in *C. cassiicola*, we generated constructs containing the endosomal marker mCherry-CcRab52 [31,39] and the TGN marker CcKex2-mCherry [17,40]. These constructs were co-transformed with CcVps35-GFP into the Δ*Ccvps35*, respectively. This revealed that CcVps35-GFP partially co-localized with mCherry-CcRab52 and CcKex2-mCherry in hyphal cells (Figure 5b,c). To assess the localization of CcVps35, the membrane-selective dye FM4-64 was used to stain the hyphae of the CcVps35-GFP strain. Our data indicated that CcVps35-GFP significantly co-localized with FM4-64-labeled vacuole membranes in hyphal cells (Figure 5a). Interestingly, CcVps29 and CcVps26 were both co-localized to the vacuolar membrane (Figure S11). Overall, these findings indicate that the retromer complex is mainly localized at the vacuolar membrane and partially to endosomes and the TGN.

### 3.6. CcSnc1 Was Cargoes for Retromer-Mediated Trafficking Pathway

**Figure 6:**
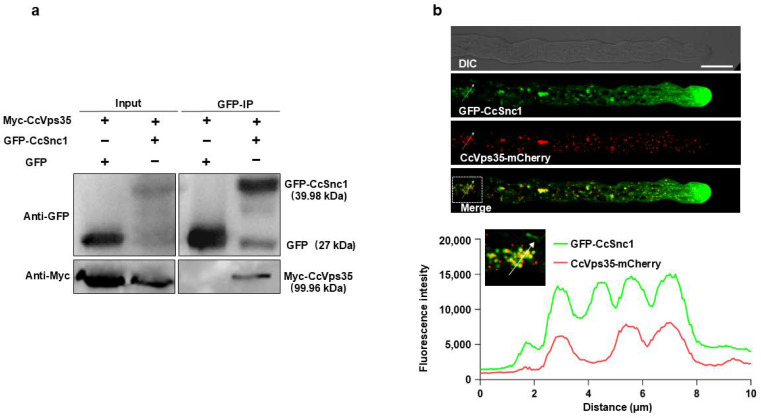
CcVps35 interacts with CcSnc1. (**a**) GFP-trap-based pull-down experiment indicating the interaction between Myc-CcVps35 and GFP-CcSnc1 in the transformant. A strain co-producing the indicated proteins was used. The GFP-CcSnc1 protein was immunoprecipitated using GFP-trap beads. The total protein extracts and IP fractions were analyzed by Western blotting. The IP signal (GFP-CcSnc1) and Co-IP signal (CcVps35-Myc) were detected by immunoblotting using anti-GFP and anti-Myc antibodies, respectively. (**b**) Representative confocal micrographs showing partial co-localization (in yellow; arrowheads) between GFP-CcSnc1 and CcVps35-mCherry in vegetative hyphae. DIC: differential interference contrast. The white dotted box represents the measurement position. Scale bar 10 μm.

The retromer complex plays a significant role in facilitating the retrograde trafficking of specific cargo proteins from plasma membrane/endosomes to the TGN in various organisms [35,41,42]. We investigated potential cargoes involved in the pathway in *C. cassiicola*. One candidate of interest protein, CcSnc1 (BS50DRAFT_576238), shared high homology with that of the *Saccharomyces cerevisiae* [22]. The 3D structures of these proteins were predicted by A_LPHA_F_OLD2_. CcSnc1 encodes a polypeptide of 120 amino acids with transmembrane domains at positions 95–117 (Figure S12). Snc1 regulates the fusion of secretory vesicles with the plasma membrane in yeast, and the homologs of Snc1 have similar functions in *M. oryzae* and *F. graminearum* [16,31,43]. Despite these findings, the functional relationship between CcSnc1 and the retromer-mediated vesicle trafficking pathway remained unestablished. Therefore, we performed a Co-IP assay to test the interaction between CcSnc1 and CcVps35 in vivo. We used a *C. cassiicola* strain producing GFP-CcSnc1 and CcVps35-Myc, and we immunoprecipitated the CcSnc1-GFP protein from extracted cellular proteins using anti-GFP beads. After immunoprecipitation of CcSnc1-GFP, detection with an Myc antibody revealed the presence of CcVps35-Myc in the immunoprecipitated fraction, suggesting the interaction between CcSnc1 and CcVps35 (Figure 6a). The subcellular localization assay further confirmed that CcVps35 and CcSnc1 shared similar localization in hyphal cells (Figure 6b). The Co-IP and co-localization assays demonstrated a close interaction between CcVps35 and CcSnc1 in *C. cassiicola*.

**Figure 7:**
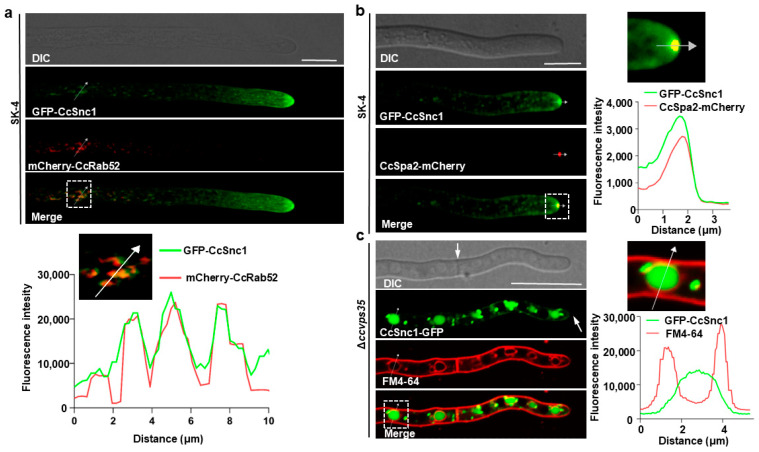
The CcVps35 plays a critical role in the proper localization of CcSnc1. (**a**,**b**) GFP-CcSnc1 co-localizes with the endosomes marked by mCherry-CcRab52 and with the apex marked by CcSpa2-mCherry in vegetative hyphal. White arrows indicate the co-localization sites. (**c**) Hyphae of the strain Δ*Ccvps35* producing CcVps35-GFP were stained with the endocytic dye FM4-64; confocal micrographs showed that the *CcVPS35* gene deletion disrupted the normal localization of CcSnc1, leading to its abnormal sorting toward degradative vacuoles. White arrows indicate the co-localization sites. DIC: differential interference contrast. The white dotted box represents the measurement position. Scale bar 10 μm.

The subcellular localization of CcSnc1 was also investigated in hyphal cells. The polarisome marker CcSpa2-mCherry [30,31], the endosomal marker mCherry-CcRab52, and the TGN marker CcKex2-mCherry encoding plasmids were co-transformed with the GFP-CcSnc1 encoding plasmid into the protoplast of Δ*Ccsnc1*. Confocal microscopic examination indicated that CcSnc1 mainly localized to polarisome and partially to endosomes or to endosomal membranes (Figure 7a,b and Figure S13). Interestingly, GFP-CcSnc1 was also found to localize on the plasma membrane and septum in hyphal cells (Figure S14). Furthermore, time-lapse microscopy showed continuous movement of GFP-CcSnc1 from endosomes toward the hyphal tips, a pivotal mechanism driving polar hyphal growth (Video S1). Therefore, we hypothesized that this dynamic transport of CcSnc1 occurs via the retromer-mediated trafficking pathway. To validate our hypothesis, we assessed the subcellular distribution of GFP-CcSnc1 in Δ*Ccvps35* after FM4-64 staining. The absence of *CcVPS35* was shown to inhibit the transport of GFP-CcSnc1 to the plasma membrane, septa, or hyphal apex in the results. Instead, GFP-CcSnc1 was redirected to the vacuolar degradation pathway (Figure 7c). These results indicate that GFP-CcSnc1 is sorted and transported via the retromer-mediated trafficking pathway, which is critical for the polar growth of *C. cassiicola*. Additionally, phylogenetic analysis revealed that both CcSnc1 and its SNARE domains are highly conserved in fungi (Figures S12 and S15).

**Figure 8:**
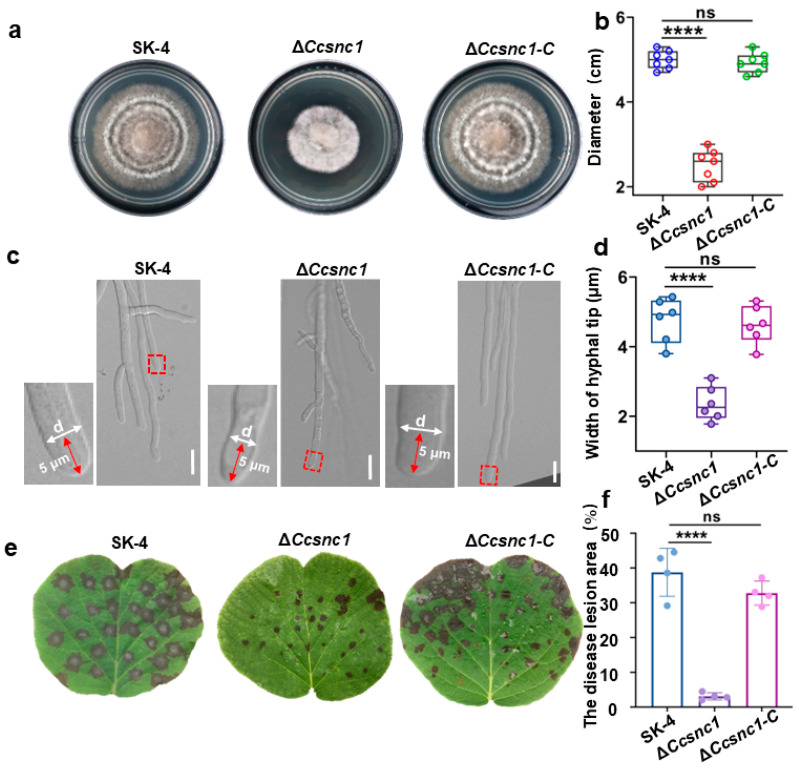
*CcSnc1* contributes significantly to the vegetative growth and virulence of *C. cassiicola*. (**a**) Colonies of the wild-type (SK-4), *CcSNC1* deletion (Δ*Ccsnc1*) and complemented strain (Δ*Ccsnc1-C*) cultured on PDA at 28 °C for 7 days. (**b**) Quantification of the colony growth rate of SK-4, Δ*Ccsnc1* and Δ*Ccsnc1-C* cultured on PDA at 7 dpi. (**c**) Hyphal tip growth and branching patterns of SK-4, Δ*Ccsnc1* and Δ*Ccsnc1-C* strains on PDA. Δ*Ccsnc1* showed significant defects in polarized growth and cell expansion at the hyphal tip. The red arrow represents 5μm from the hypha tip. The red dotted box represents the measurement position. “d” represents the diameter of hyphae. (**d**) Quantification of the width of hyphae measured 5 µm from the tip in strains SK-4, Δ*Ccsnc1* and Δ*Ccsnc1-C*. (**e**) Images of conidium-infected kiwifruit leaves. (**f**) Quantification of the pathogenicity assay results. The disease lesion area (%) indicated the percentage of lesion size area to total leaf area. The values shown are the means of independent experiments. Statistical analysis was processed by one-way ANOVA for multiple comparisons using GraphPad Prism 9 (**** *p* < 0.0001, ns, not significant at *p* > 0.05). Scale bar 10 μm.

A recent study demonstrated that Snc1 was involved in the development and virulence of *F. graminearum* [31]. To investigate its biological role in *C. cassiicola*, we generated *CcSNC1* deletion (Δ*CcSNC1*) and confirmed the deletion by PCR and qRT-PCR (Figure S16). The deletion mutants displayed defects in growth, development, and pathogenicity, which were similar to those of the retromer complex mutants (Figure 8). These results suggest that the retromer is essential in managing the transport of cargo protein CcSnc1 in *C. cassiicola*, ensuring its continuous delivery to both the hyphal tip and plasma membrane, a process that intricately regulates the growth, development, and pathogenicity of *C. cassiicola*.

## 4. Discussion

**Figure 9:**
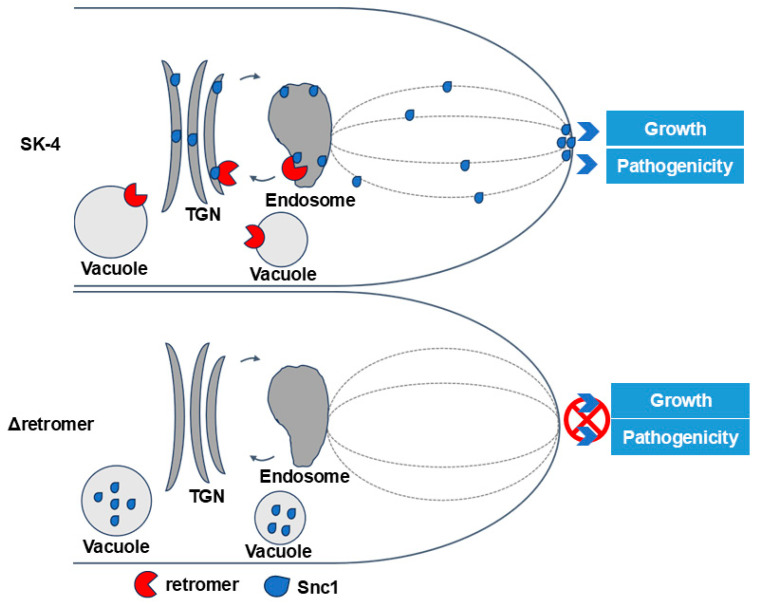
A proposed model of the retromer complex module-mediated vesicle trafficking pathway and functions in *C. cassiicola*. In the wild-type SK-4, the retromer regulates retromer-mediated vesicles retrograde transport from endosomes to the TGN. Several cargo proteins, like Snc1, on the endosome are recognized by the retromer complex and recycled through module-mediated vesicular transport pathway to the TGN, which functions in endocytic recycling, including plasma membrane to hyphal tips. In the retromer complex null mutant, the vesicular retrograde trafficking pathway is greatly impaired, leading to Snc1 being transported to the vacuole, and this results in the vacuolar degradation of the proteins. The function of the retromer complex is to recruit CcSnc1 for retrieval, sorting, and transport from the endosome membrane to the TGN membrane. Loss of the retromer-mediated vesicle-trafficking pathway in *C. cassiicola* leads to impairment of hyphal growth and pathogenicity.

In eukaryotes, vesicle transport is essential for ensuring specific cargo proteins reach their destinations to maintain normal cellular activities [13]. The retromer complex, a pivotal component, facilitates the transport of various cargo proteins from endosomes to the TGN, and it is crucial to uphold cellular physiological functions [35,44,45]. While homologs of the retromer complex demonstrate diverse functions in different eukaryotes, they are essential for fungal growth, development, and pathogenicity [17,18,46,47]. In this study, we investigated the role of the retromer complex in *C. cassiicola* for the first time. The growth rate and pathogenicity of deletion mutants of the retromer complex were decreased significantly. Our findings indicate that the retromer complex regulates growth, development, and pathogenicity by orchestrating the transport of the cargo protein CcSnc1 to the hyphal tips and various organelles during *C. cassiicola* invasion (Figure 9).

The retromer complex is essential for enabling the retrograde transport of cellular cargo proteins from endosomes to the trans-Golgi network (TGN) [35]. Malfunction or loss of the retromer complex can result in the mistargeting of cargo proteins, further leading to various pathological conditions [48,49,50]. As a core component of the retromer complex, Vps35 directly participates in the sorting and transport of cargo proteins [51,52]. *CcVps35*, *CcVps29* and *CcVps26*, found to be relevant for pathogenicity, based on transcriptome analysis, share high homology to those from yeast (Figures S7–S9). Further gene function analysis showed that *CcVps35*, *CcVps29* and *CcVps26* are essential for the growth, development, and pathogenicity of *C. cassiicola* (Figure 4). The functional role of the retromer complex has been reported in yeasts, plants, animals and some fungi [17,18,46,47], but the function remains unclear in *C. cassiicola*. To elucidate the molecular mechanisms involved, we conducted subcellular co-localization analyses of CcVps35, CcVps29 and CcVps26, revealing the localization of them to vacuoles, endosomes, and the TGN (Figure 5 and Figure S11). Additionally, the subunits of the retromer complex strongly interacted with each other by yeast two-hybrid assay (Figure 3). Previous studies have demonstrated that the retromer complex facilitates the transport of cargo proteins, such as Vps10, from endosomes to the Golgi apparatus, regulating cellular functions in both yeast and *Arabidopsis* [53,54], but it has not been reported in *C. cassiicola* so far. To further explore how the retromer complex regulates the biological functions of cargo proteins, we investigated the cellular localization of CcSnc1 in the Δ*Ccvps35* mutant and found that loss of the *CcVPS35* resulted in the miscarriage of CcSnc1 to vacuolar degradation pathways. This revealed that CcSnc1 was a cargo protein in the retromer complex transport pathway (Figure 7). Thus, we detected the interaction between the core subunit CcVps35 and the cargo protein CcSnc1 by co-localization and Co-IP assay (Figure 6). We found intensive interaction between CcVps35 and CcSnc1. Furthermore, we knocked out the *CcSNC1*, and the lack of *CcSNC1* led to defects in growth, development, and pathogenicity of *C. cassiicola*, suggesting that CcSnc1 is crucial for the infection of *C. cassiicola* (Figure 8). Therefore, we conclude that the retromer complex regulates the growth, development, and pathogenicity of *C. cassiicola* by mediating the transport of the cargo protein CcSnc1. However, the processes by which the retromer complex accurately sorts cargo proteins to endosomes, further coordinates their cycling to the TGN, and affects growth, development, and pathogenicity deserves further study in C. *cassiicola*.

In addition, transcriptome analysis showed that the early stages of *C. cassiicola* infection caused significant changes in the expression of a number of genes associated with vesicular transport, especially those related to vesicular transport responsible for effector secretion. These small and cysteine-rich proteins are released by pathogens to suppress plant immunity and promote colonization [55,56]. For the translocation of effectors, the secretion pathway mediated by retromer complexes in *C. cassiicola* remains unclear. To date, the effector transport pathway was thoroughly investigated in *M. oryzae*, which was divided into the conservative secretion pathway and the unconventional protein secretion (UPS) pathway [57]. Effectors such as LysM protein 1 and Bas4 are transported into the apoplastic compartment and enclosed by extensions of the plant plasma membrane known as the extra-invasive hyphal membrane (EIHM), which uniformly outlines the entire invasive hyphae (IH) through the conservative secretion pathway [58,59]. Cytoplasmic effectors (AvrPita, Avr-Pizt, Pwl1 and Pwl2) secreted via the UPS pathway firstly gather in a novel membrane structure known as the biotrophic interfacial complex (BIC) and are subsequently transported into host cells through the exocyst complex and MoSso1-mediated secretion [60]. Despite the growing evidence suggesting that the retromer complex plays a crucial role in endosome–plasma membrane protein trafficking, direct proof concerning free retromer vesicles as transport vectors at the endosome–plasma membrane interface remains elusive, particularly during pathogen–host interactions. Therefore, subsequent experiments will focus on the secretion transport of *C. cassiicola* effectors, as regulated by vesicle transport.

## Data Availability

The original data presented in the study are openly available in NCBI at https://www.ncbi.nlm.nih.gov/sra/PRJNA1156053 (accessed on 15 September 2024).

## Supplementary Materials

The following supporting information can be downloaded at: https://www.mdpi.com/article/10.3390/jof10100714/s1, Figure S1: The *CcVPS35* gene replacement strategy; Figure S2: The *CcVPS29* gene replacement strategy; Figure S3: The *CcVPS26* gene replacement strategy; Figure S4: Observation of lesion changes and mycelial morphology during *C. cassiicola* infection; Figure S5: The 20 most enriched KEGG pathways in *C. cassiicola*; Figure S6: The comparison of gene expression levels obtained by qRT-PCR and RNA-seq at 24 hpi, 72 hpi; Figure S7–S9: Phylogenetic analysis of putative Vps35/26/29 orthologs in fungi, plants and human; Figure S10: Localization of CcVps35-GFP; Figure S11: Localization of CcVps29 and CcVps26 in *C. cassiicola*; Figure S12: The protein structure of CcSnc1 predicted with ALPHAFOLD2; Figure S13: Co-localization of CcKex2-mCherry and GFP-CcSnc1; Figure S14: Localization of GFP-CcSnc1; Figure S15: Phylogenetic analysis of putative Snc1 orthologs in fungi; Figure S16: The *CcSNC1* gene replacement strategy; Video S1: Dynamics and mobility of GFP-CcSnc1 in growing hyphae of *C. cassiicola*; Table S1: The fungal strains used in this study; Table S2: RT-qPCR primers used in this study; Table S3: PCR primers used in this study; Table S4: The plasmids used in this study; Table S5: Summary of Illumina sequencing and transcriptome assemblies for RNA-Seq libraries; Table S6: The data of RT-qPCR and RNA-seq at 24 hpi and 72 hpi.
