# Supplementary material for: The Sorting and Transport of the Cargo Protein CcSnc1 by the Retromer Complex Regulate the Growth, Development, and Pathogenicity of Corynespora cassiicola
Source: J Fungi (Basel). 2024 Oct 14;10(10):714. doi: 10.3390/jof10100714 (PMC11508248; doi:10.3390/jof10100714)
Supplement: Supplementary file 1 [file jof-10-00714-s001.zip › jof-3172391-supplementary.pdf]

Supplemental Material

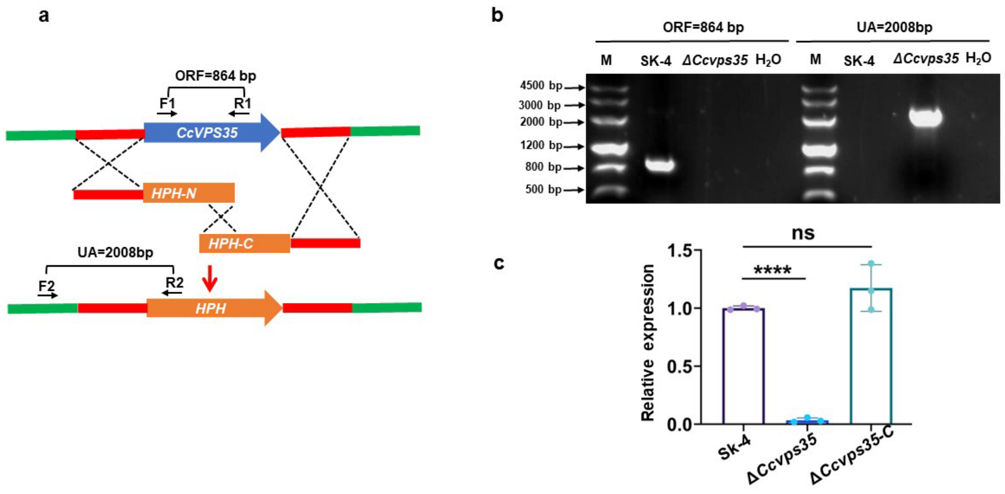

Figure S1. The *CcVPS35* gene replacement strategy. (a) *CcVPS35* gene replacement strategy is shown in the schematic diagram. The primer pairs F1/R1 and F2/R2 were used for mutant screening and identification. (b) PCR analysis of SK-4 and *CcVPS35* deletion mutant was performed using genomic DNA as template. ORF showed a 0.864 kb band in the SK-4 and a 2.008 kb band in the mutants, confirming that the *ΔCcvps35* generated is the right mutant. (c) Expression levels of *CcVPS35* in WT, Δ*Ccvps35* and Δ*Ccvps35*-C strains was monitored with qRT-PCR. The Actin gene as the endogenous reference. M: molecular markers; SK-4: WT strain; H_2O: negative control, Statistical analysis was processed by one-way ANOVA for multiple comparisons using GraphPad Prism 9 (****p < 0.0001).

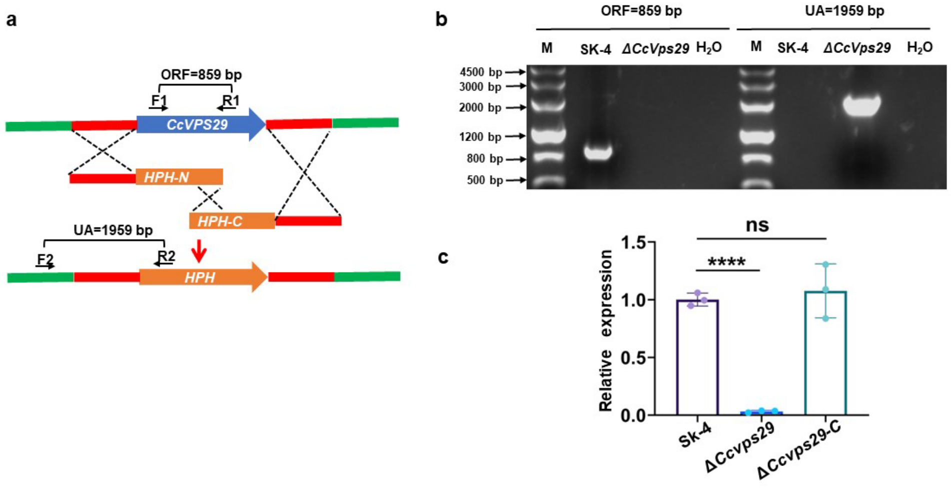

Figure S2. The *CcVPS29* gene replacement strategy. (a) *CcVPS29* gene replacement strategy is shown in the schematic diagram. The primer pairs F1/R1 and F2/R2 were used for mutant screening and identification. (b) PCR analysis of *CcVPS29* gene deletion mutant was

performed using genomic DNA as template. ORF showed a 0.859 kb band in the SK-4 and a 1.959 kb band in the mutants, confirming that the *ΔCcvps29* generated is the right mutant. (c) Expression levels of *CcVPS29* in WT, Δ*Ccvps29* and Δ*Ccvps29*-C strains was monitored with qRT-PCR. The Actin gene as the endogenous reference. M: molecular markers; SK-4: WT strain; H_2O: negative control, Statistical analysis was processed by one-way ANOVA for multiple comparisons using GraphPad Prism 9 (****p < 0.0001).

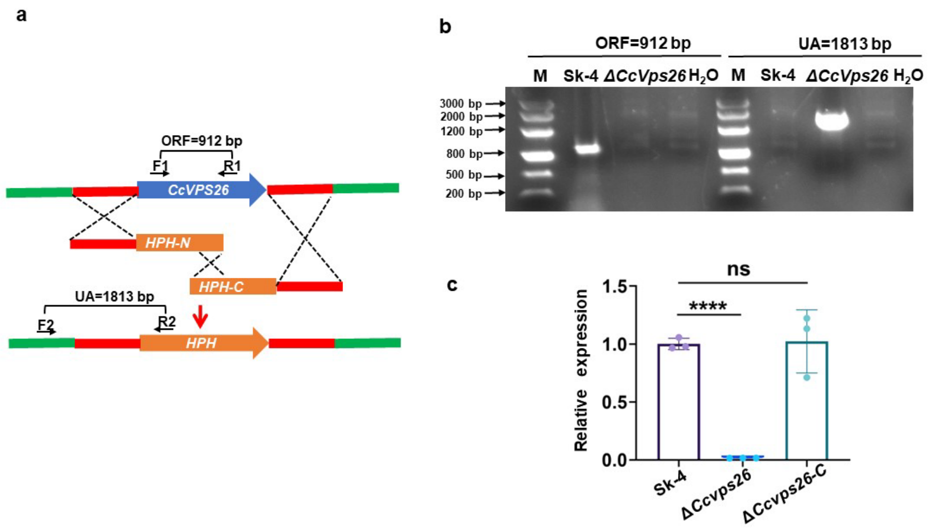

Figure S3. The *CcVPS26* gene replacement strategy. (a) *CcVPS26* gene replacement strategy is shown in the schematic diagram. The primer pairs F1/R1 and F2/R2 were used for mutant screening and identification. (b) PCR analysis of *CcVPS26* gene deletion mutant was performed using genomic DNA as template. ORF showed a 0.912 kb band in the SK-4 and a 1.813 kb band in the mutants, confirming that the *ΔCcvps26* generated is the right mutant. (c) Expression levels of *CcVPS26* in WT, Δ*Ccvps26* and Δ*Ccvps26*-C strains was monitored with qRT-PCR. The Actin gene as the endogenous reference. M: molecular markers; SK-4: WT strain; H_2O: negative control, Statistical analysis was processed by one-way ANOVA for multiple comparisons using GraphPad Prism 9 (****p < 0.0001).

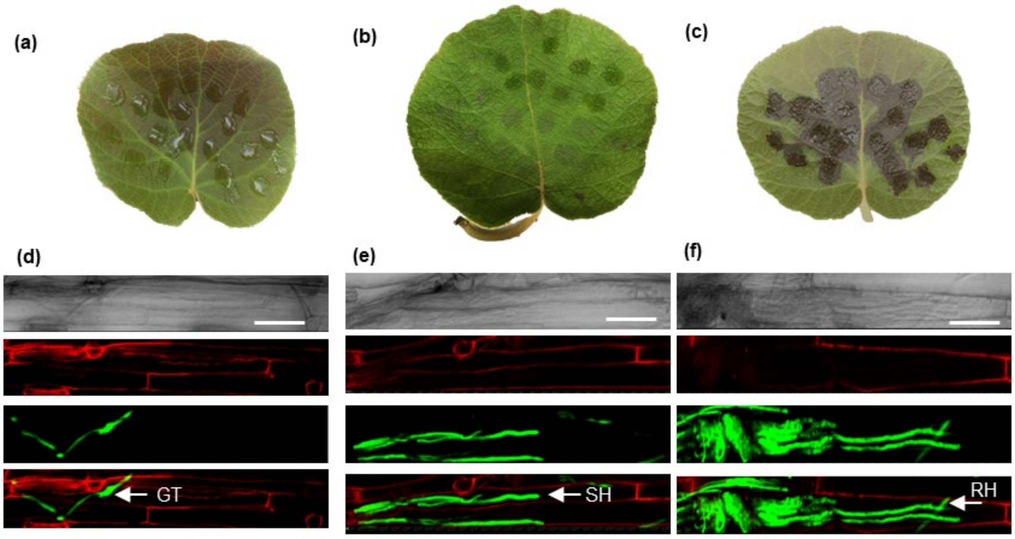

Figure S4. Observation of lesion changes and mycelial morphology during *C.cassiicola* infection. (a-c) Observation of symptoms after inoculation with *C.cassiicola* at 0 h, 24 h and 72 h, respectively. (d-e) Confocal micrographs showing the elongation of vegetative hyphae after inoculation with *C.cassiicola* at 3 h, 24 h and 72 h, respectively. GT: germ tubes; SH: superficial hyphae; RH: radiating hyphae. White arrows indicate the hyphae tip continues to extend forward. Scale bars 10 µm.

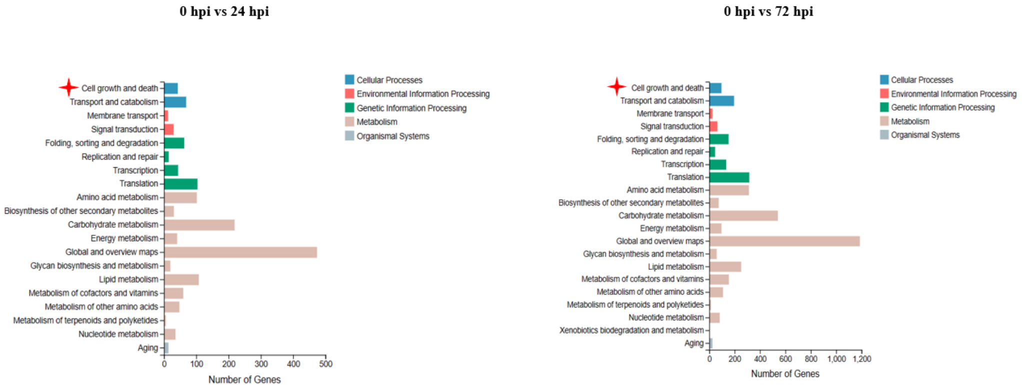

Figure S5. The 20 most enriched KEGG pathways in C.cassiicola. The 20 most enriched KEGG pathways iof *C.cassiicola* during infection at 24 h (left) and 72 h (right). Asterisks denote the presence of Cell growth and death among KEGG pathways.

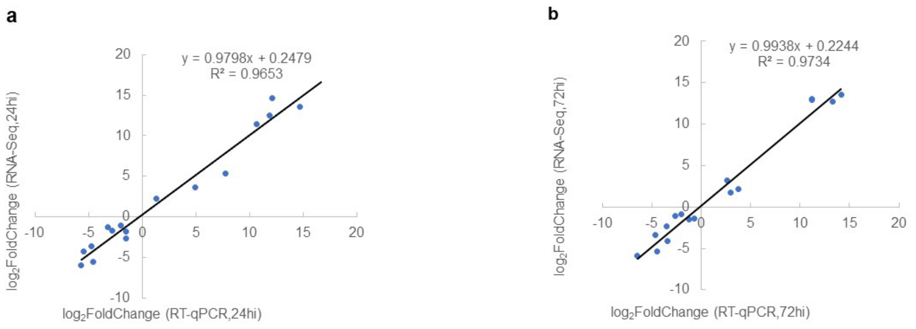

Figure S6. The comparison of gene expression levels obtained by qRT-PCR and RNA-seq at 24 hpi, 72hpi. (a) 0 hpi vs 24 hpi; (b) 0 hpi vs 72 hpi. qRT-PCR : The gene expression $\log_2$(Fold Change) value of 16 differentially expressed genes (DEGs) in qRT-PCR analysis; RNA-seq: The gene expression $\log_2$(Fold Change) value of DEGs in RNA-seq analysis, The Actin gene as the endogenous reference.

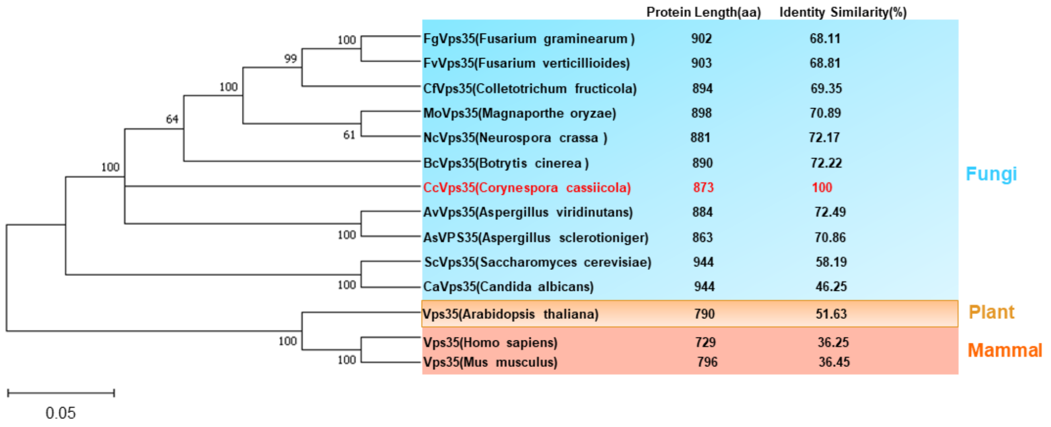

Figure S7. Phylogenetic analysis of putative Vps35 orthologs in fungi, plants and human. A neighbor-joining tree was constructed based on the amino acid sequences. The number at nodes represent the percentage of their occurrence in 1,000 bootstrap replicates. GenBank accession numbers (corresponding species names) of Vps35 orthologs are as follows: XP_011318722.1(FgVps35 *Fusarium graminearum*), XP_018755580.1 (FvVps35 *Fusarium verticillioides*), XP_031881347.1 CfVps53 (*Colletotrichum fructicola*), XP_003712611.1 (MoVps35 *Magnaporthe oryzae*), XP_962546.1 (NcVps35 *Neurospora crassa*), XP_001556673.1 (BcVPS35 *Botrytis cinerea*), PSN66776.1 (CcVps35 *Corynespora cassiicola*), XP_043124663.1 (AvVps35 *Aspergillus viridinutans*), Xp_025467599.1 (AsVps35 *Aspergillus sclerotioniger*), AJV39625.1 (ScVps35 *Saccharomyces cerevisiae*), XP_712805.1 (CaVps35 *Candida albicans*), NP_190699.3 (Vps35 *Arabidopsis thaliana*), XP_005256102.1 (Vps35 *Homo sapiens*), NP_075373.1 (Vps35 *Mus musculus*).

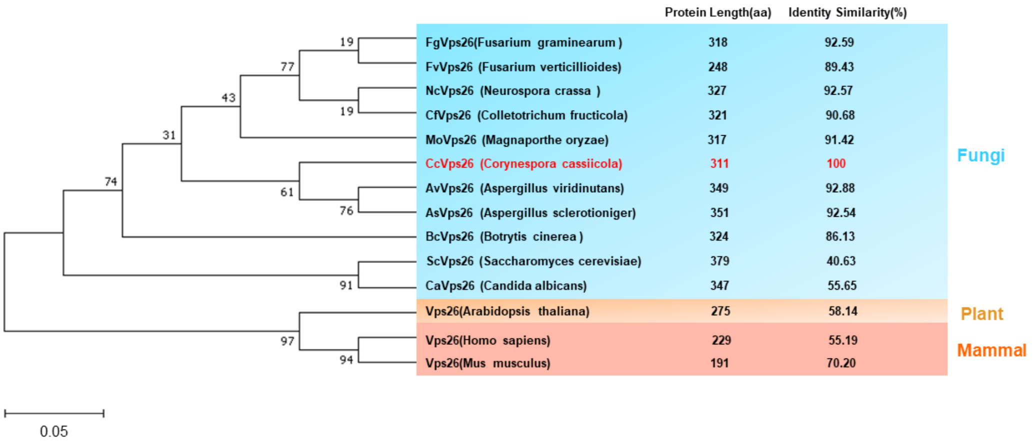

Figure S8. Phylogenetic analysis of putative Vps26 orthologs in fungi, plants and human. A neighbor-joining tree was constructed based on the amino acid sequences. The number at nodes represent the percentage of their occurrence in 1,000 bootstrap replicates. GenBank accession numbers (corresponding species names) of Vps26 orthologs are as follows: XP_011316923.1 (CcVps26 *Fusarium graminearum*), XP_965128.3 (NcVps26 *Neurospora crassa*), XP_018743988.1 (FvVps26 *Fusarium verticillioides*), XP_031879784.1 (CfVps26 *Colletotrichum fructicola*), XP_003713759.1 (MoVps26 *Magnaporthe oryzae*), PSN72773.1 (CcVps26 *Corynespora cassiicola*), XP_043126348.1 (AvVps26 *Aspergillus viridinutans*), XP_025471819.1 (AsVps26 *Aspergillus sclerotioniger*), Xp_024551650.1 (BcVPS26 *Botrytis cinerea*), NP_012482.1 (ScVps26 *Saccharomyces cerevisiae*), XP_712728.2 (CaVps26 *Candida albicans*), NP_001031732.1 (Vps26 *Arabidopsis thaliana*), AAH29758.1 (Vps26 *Homo sapiens*), XP_011540867 (Vps26 *Mus musculus*).

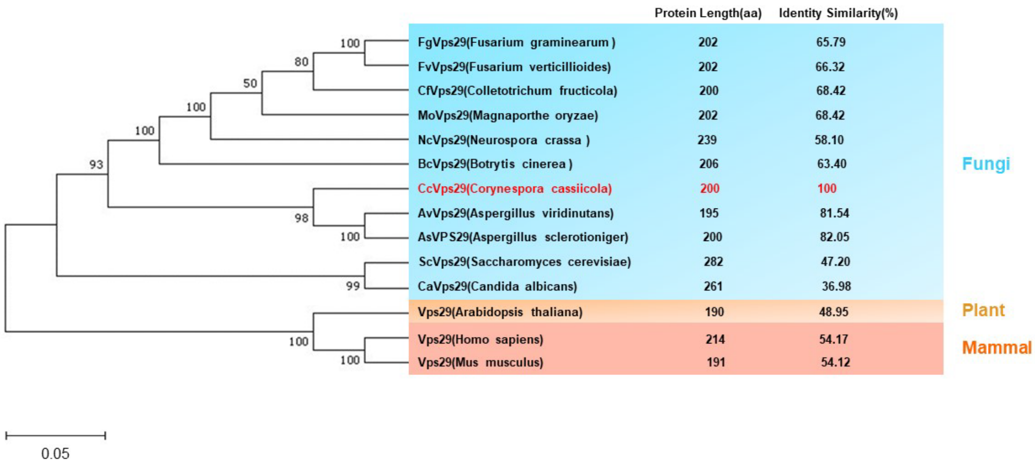

Figure S9. Phylogenetic analysis of putative Vps29 orthologs in fungi, plants and human. A neighbor-joining tree was constructed based on the amino acid sequences. The number at nodes represent the percentage of their occurrence in 1,000 bootstrap replicates. GenBank accession numbers (corresponding species names) of Vps29 orthologs are as follows: XP_011317363.1 (FgVps29 *Fusarium graminearum*), XP_018756589.1 (FvVps29 *Fusarium verticillioides*), XP_031883282.1 (CfVps29 *Colletotrichum fructicola*), XP_955919.1 (MoVps29 *Magnaporthe oryzae*), XP_003709334.1 (NcVps29 *Neurospora crassa*), Xp_024552783.1 (BcVPS29 *Botrytis cinerea*), PSN68731.1 (CcVps29 *Corynespora cassiicola*), XP_043125909.1

(AvVps29 Aspergillus viridinutans), XP_025466055.1 (AsVps29 *Aspergillus sclerotioniger*), NP_011876.1 (ScVps29 *Saccharomyces cerevisiae*), XP_719038.1 (CaVps29 *Candida albicans*), NP_190365.3 (Vps29 *Arabidopsis thaliana*), NP_001269079.1 (Vps29 *Homo sapiens*), NP_001346155.1 (Vps29 *Mus musculus*).

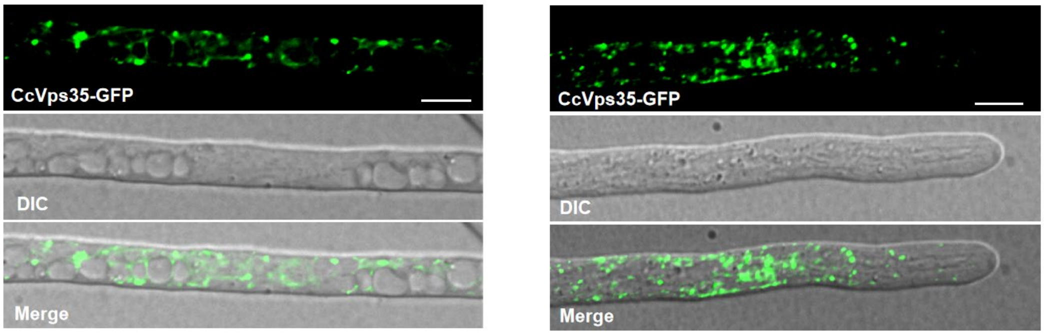

Figure S10. Localization of CcVps35-GFP. Confocal micrographs showing CcVps35 localized to cytoplasm with punctates in the middle (left) and tips (right) vegetative hyphae. DIC: Differential Interference Contrast. Scale bars 10 μm.

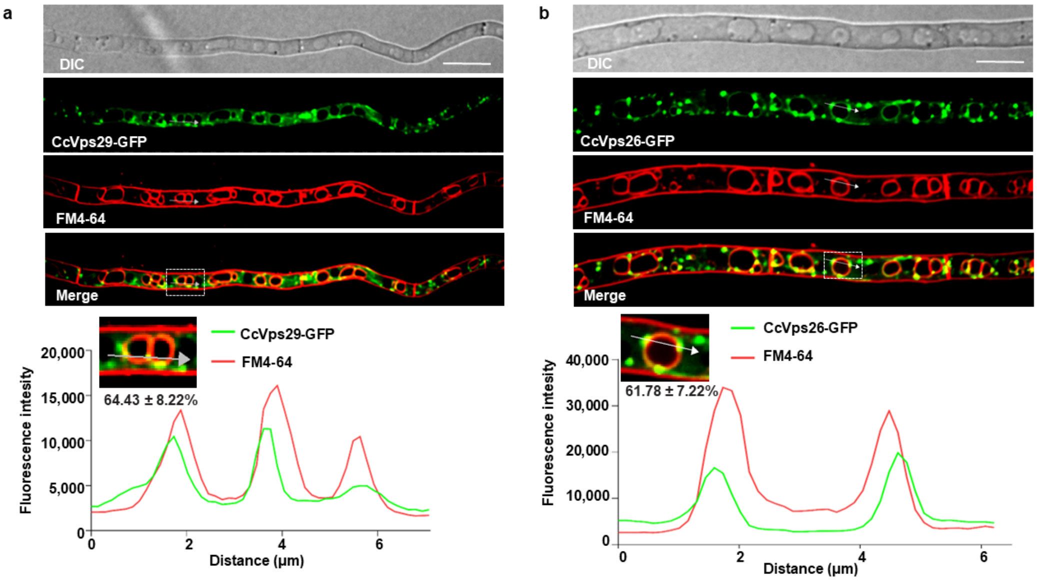

Figure S11. Localization of CcVps29 and CcVps26 in *C.cassiicola*. (a) Hyphae expressing CcVps29-GFP were stained with endocytic dye FM4-64 and observed under a confocal microscope. CcVps29-GFP is localized to the vacuolar membrane and partially at endosomes and the TGN, the co-localization rate is 64.43 ± 8.22%. White arrows indicate the co-localization sites. Scale bars =10 μm. (b) Hyphae expressing CcVps26-GFP were stained with endocytic dye FM4-64 and observed under a confocal microscope. CcVps26-GFP is localized to the vacuolar membrane and partially at endosomes and the TGN, the co-localization rate is 61.78 ± 7.22%. Scale bars 10 μm.

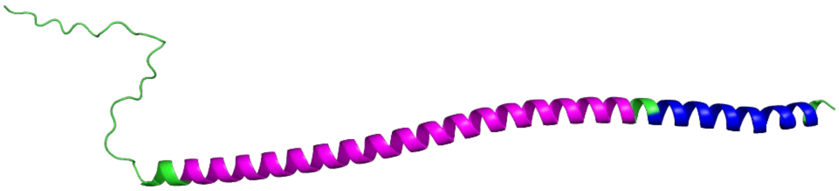

Figure S12. The protein structure of CcSnc1 predicted with ALPHAFOLD2. The conserved SNARE (residues 31-89, red) and TM (residues 95-117, blue) domains of CcSnc1 are shown.

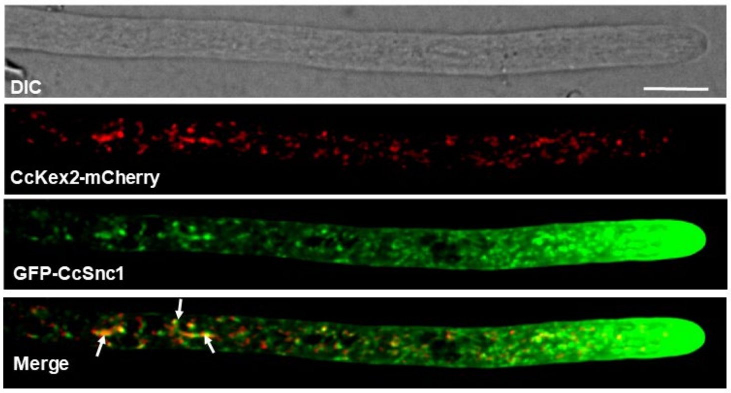

Figure S13. Co-localization of CcKex2-mCherry and GFP-CcSnc1. Co-expression of the Golgi apparatus CcKex2-mCherry and GFP-CcSnc1 showing co-localization in vegetative hyphae, White arrows indicate the co-localization sites, micrographs showing CcSnc1 is localized on the Golgi apparatus; DIC, differential interference contrast; Scale bar 10 μm.

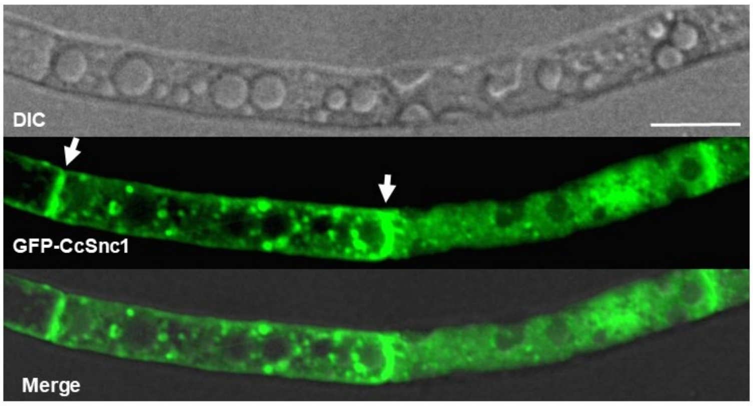

Figure S14. Localization of GFP-CcSnc1. Confocal micrographs showing CcSnc1 localized on the plasma membrane and septum in hyphal cells. White arrows indicate the plasma membrane and septum in hyphal cells. DIC, differential interference contrast; Scale bars 10 μm.

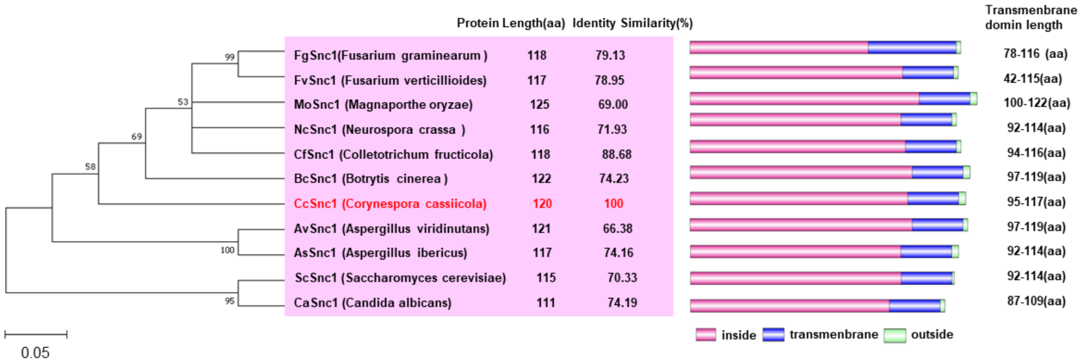

Figure S15. Phylogenetic analysis of putative Snc1 orthologs in fungi. A neighbor-joining tree was constructed based on the amino acid sequences. The number at nodes represent the percentage of their occurrence in 1,000 bootstrap replicates. GenBank accession numbers (corresponding species names) of Snc1 orthologs are as follows: XP_011320221.1 (FgSnc1 *Fusarium graminearum*), XP_018745560.1 (FvSnc1 *Fusarium verticillioides*), XP_964727.1 (NcSnc1 *Neurospora crassa*), XP_001554594.1 (BcSnc1 *Botrytis cinerea*), XP_031880466.1 (CfSnc1 *Colletotrichum fructicola*), PSN63609.1 (CcSnc1 *Corynespora cassiicola*), XP_043129457.1 (AvSnc1 *Aspergillus viridinutans*), XP_025571197.1 (AsSnc1 *Aspergillus ibericus*), XP_003721079.1 (MoSnc1 *Magnaporthe oryzae*), NP_014972.3 (ScSnc1 *Saccharomyces cerevisiae*), XP_019330726.1 (CaSnc1 *Candida albicans*).

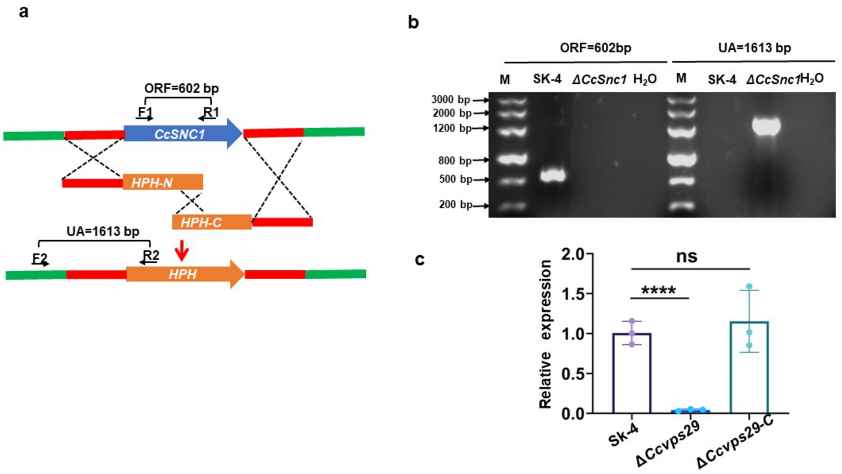

Figure S16. The *CcSNC1* gene replacement strategy. (a) *CcSNC1* gene replacement strategy is shown in the schematic diagram. The primer pairs F1/R1 and F2/R2 were used for mutant screening and identification. (b) PCR analysis of *CcSNC1* gene deletion mutant was performed using genomic DNA as template. ORF showed a 0.602 kb band in the SK-4 and a 1.613 kb band in the mutants, confirming that the *ΔCcsnc1* generated is the right mutant. (c) Expression levels of *CcSNC1* in WT, Δ*Ccsnc1* and Δ*Ccsnc1*-C strains was monitored with qRT-PCR. The Actin gene as the endogenous reference. M: molecular markers; SK-4: WT strain; H_2O: negative control, Statistical analysis was processed by one-way ANOVA for multiple comparisons using GraphPad Prism 9 (****p < 0.0001).

Video S1. Dynamics and mobility of GFP-CcSnc1 in growing hyphae of *C. cassiicola*. Small punctae in growing hyphae of GFP-CcSnc1 co-localization with mCherry-CcRab52 move very fast from endosomes towards hyphal tips.

Table S1. The fungal strains used in this study.

Strain	Genotype description	Reference
SK-4	Wild-type	Cuomo *et al.* (2007)
ΔCcvps35	*CcVPS35* deletion mutant of Sk-4	This study
ΔCcvps35-C	ΔCc*vps35* transformant expressing CcVps35-GFP construct	This study
CcVps35-GFP+mCherry-CcRab52	Sk-4 transformant expressing both CcVps35-GFP and mCherry-CcRab52 constructs	This study
CcVps35-GFP+CcKex2-mCherry	Sk-4 transformant expressing both CcVps35-GFP and CcKex2-mCherry constructs	This study
ΔCcvps29	*CcVPS29* deletion mutant of Sk-4	This study
ΔCcvps29-C	ΔCc*vps29* transformant expressing CcVps29-GFP construct	This study
ΔCcvps26	*CcVPS26* deletion mutant of Sk-4	This study
ΔCcvps26-C	ΔCc*vps26* transformant expressing CcVps29-GFP construct	This study
ΔCcSnc1	*CcSnc1* deletion mutant of Sk-4	This study
ΔCcSnc1-C	ΔCcsnc1 transformant expressing GFP-CcSnc1 construct	This study
GFP-CcSnc1+mCherry-CcRab52	Sk-4 transformant expressing both GFP-CcSnc1 and mCherry-CcRab52 constructs	This study
GFP-CcSnc1+CcKex2-mCherry	Sk-4 transformant expressing both GFP-CcSnc1 and CcKex2-mCherry constructs	This study
GFP-CcSnc1+CcSpa2-mCherry	Sk-4 transformant expressing both GFP-CcSnc1 and CcSpa2-mCherry constructs	This study
ΔCc*vps35*+GFP-CcSnc1	ΔCc*vps35* transformant expressing GFP-CcSnc1 constructs	This study

Table S2. RT-qPCR primers used in this study.

primers	primer sequence（5′-3′）	fragment size/bp
KWCC_176962-F	GACAGAACGCCATGAACGC	113
KWCC_176962-R	CGGAAACAAGCAGCAACAGG	
KWCC_479264-F	CCCCAGGACAATAAGAATG	109
KWCC_479264-R	CGCCTCGTAGGATGGAACT	
KWCC _538366-F	GCAGCCCTTACCCTCCTAC	130
KWCC _538366-R	TGACCAGCGAAGTGACAGA	
KWCC _569508-F	CTTGCCGTCACCCTATTCT	148
KWCC _569508-R	CTCCCATTTCCTTGCCACC	
KWCC _574302-F	ACAAGTCGCAATGGACAACC	122
KWCC _574302-R	GAAAGAGGACCAGACGAAGC	
KWCC _584320-F	ATTTCCTGTTGGTGGTTATCT	102
KWCC _584320-R	GCACTACTCCGAACGAGCT	
KWCC _604234-F	AATGGAGCGAAGTTGCGGGTAT	106
KWCC _604234-R	TGAGGTGCGGCGGGTTGTAG	
KWCC _613819-F	TGCGTGGGTATCTTCCGTGAT	151
KWCC _613819-R	CGGTGTTGGCAGGGTTCAT	
KWCC _629187-F	AGACCCTGGAAGCGAATCCT	111
KWCC _629187-R	GGCTGGCGACAAAGTTACCC	
KWCC _635126-F	GTCGGCGTTGACCTCTGTG	120
KWCC _635126-R	CGCTTCAATCTCGGCAAAA	
KWCC _637300-F	TCGCAGAAGCCCGTGACTC	119
KWCC _637300-R	GCGTGCTCAAACACCTCCC	
KWCC _674345-F	AAAGGTCATCCGAACTCAC	130
KWCC _674345-R	TGTATTCAAATGCTGTGGC	
KWCC _675241-F	TCATTGGGAAGTCGGTTAC	115
KWCC _675241-R	CTCGTCCTGAAGTTTCTCATC	
KWCC _573581-F	AGTTCCAGGCTGTATGCGTGAT	133
KWCC _573581-R	CGGCACTGGTCTGGCTTCT	
KWCC _652902-F	GCCCAATCAGTACAACGAGA	106
KWCC _652902-R	GAAACCGCCCAGGAACAGG	
KWCC _490648-F	CGCCGGACCTGCAAATCGT	108
KWCC _490648-R	GCCCGTGCGTGAAGCCTAT	
KWCC _Actin-F	GCGACCGACAATGGAAGCT	131
KWCC _Actin-R	CGATGCCCGCCAGCAAAGC	

Table S3. PCR primers used in this study.

Primers	Sequence (5'-3')	Application
CcVPS35AF	CAACGATCGCTTGTCGCCC	CCVPS35deletion and probe
CcVPS35AR	TTGACCTCCACTAGCTCCAGCCAAGCCGGGGTATTTCCGGGCTGG	
CcVPS35BF	GAATAGAGTAGATGCCGACCGCGGGTTACCTCAACGGACTCATCGAG	
CcVPS35BR	ATCTATCCATAACCGCGGT	
CcVPS35OF	CGACTGCAGCACCAAGGA	ΔCcVps35 mutant screen
CcVPS35OR	GCGAATTCCAAAACCTGATC	
CcVPS35UA	AACAGCTCACGCCGGAGA	
H853	GACAGACGTCGCGGTGAGTT	
CcVPS35CF (GFP)	agggaacaaaagctgggtaccCAACGATCGCTTGTCGCCC	For generation of pCcVps35-GFP
CcVPS35CR (GFP)	gcccttgctcaccataagcttTTTTGGCGCTGTCACGACA	
CcVPS29AF	CAGATGTCCCATGCGGCAT	*CCVPS29*deletion and probe
CcVPS29AR	TTGACCTCCACTAGCTCCAGCCAAGCCCGTCCCTAGGTTTGTATGGA	
CcVPS29BF	GAATAGAGTAGATGCCGACCGCGGGTTGCTGCACGTGATAGCAATTT	
CcVPS29BR	ATACATGCATAGCCTGGCCC	
CcVPS29OF	CCGATAGAGCTCCGGTAAGA	ΔCc*vps29* mutant screen
CcVPS29OR	GGTAGACGTACAGGACGAG	
CcVPS29UA	ACGAAGGTAAACAATGGCCG	
CcVPS29CF(GFP)	agggaacaaaagctgggtaccGTCGGACAGGCCTTCTGC	For generation of pCcVps29-GFP
CcVPS29CR(GFP)	gcccttgctcaccataagcttTGATACACCAGCGCCGTTT	
CcVPS26AF	CGGCGTTTGAATGTTAGCTT	*CCVPS26* deletion and probe
CcVPS26AR	TTGACCTCCACTAGCTCCAGCCAAGCCCGTCGTCGTCGTCGTCGG	
CcVPS26BF	TTGACCTCCACTAGCTCCAGCCAAGCCCGTCGTCGTCGTCGTCGG	
CcVPS26BR	GGAGCGGCGGTATCGAGGA	
CcVPS26OF	CTCGAGGACACCGACGAC	ΔCc*vps26* mutant screen
CcVPS26OR	GTGGGGTTACACACCTTCG	
CcVPS26UA	CCTGTCAGCATCCTGGCA	
CcVPS26CF(GFP)	agggaacaaaagctgggtaccAAGTTTGTGGAAAGGCGGC	For generation of pCcVps26-GFP, pFgVps53-mCherry
CcVPS26(GFP)	gcccttgctcaccataagcttTGATGCTGCCATCAACTTGT	

CcSnc1AF	GTGCTTGGTGTCTTGTGCG	*CcSNC1*deletion and probe
CcSnc1AR	TTGACCTCCACTAGCTCCAGCCAAGCCCGAGAATGCGACAGTGGTC	
CcSnc1BF	GAATAGAGTAGATGCCGACCGCGGGTTTCAGCCACGAAGGTGCCAG	
CcSnc1BR	AATTGGTGAGGCAGAGGAGA	
CcSnc1OF	CAAGACCCCCCCTACGAC	Δ*CcSNC1* mutant screen
CcSnc1OR	TGATGATGCCGACGATGAG	
CcSnc1UA	CCAAGGTCAAGCAGTGGC	
CcSnc1NF	agggaacaaaagctgggtaccGAGCGGAAGAGCGCTTTAGG	For generation of pGFP-CcSnc1 construct
CcSnc1NR	TCCTCGCCCTTGCTCACCATGTCGGCTGGAGTGGAAGA	
CcSnc1-GFPF	ATGGTGAGCAAGGGCGAGGA	
CcSnc1-GFPR	CTTGTACAGCTCGTCCATGC	
CcSnc1-CDSF	gcatggacgagctgtacaagATGGCCAGCAACGGCGCC	
CcSnc1-CDSR	tcagtaacgttaagtggatccCTATTTTTTCGTAGCAATGACTGG	
AD-CcVPS35-F	gccatggaggccagtgaattcATGGCGTCGCCGCCCCC	For generation of pAD-CcVPS35 construct
AD-CcVPS35-R	cagctcgagctcgatggatccCTATTTTGGCGCTGTCACG	
BD-CcVPS29-F	atggccatggaggccgaattcATGGCTTCCCGCCTCGTG	For generation of pAD-CcVPS35 construct
BD-CcVPS29-R	ccgctgcaggtcgacggatccCTATGATACACCAGCGCCG	
BD-CcVPS26-F	atggccatggaggccgaattcATGGCGTCGCCGCCCCC	
BD-CcVPS26-R	ccgctgcaggtcgacggatccCTATTTTGGCGCTGTCACG	
AD-CcVPS29-F	gccatggaggccagtgaattcATGGCTTCCCGCCTCGTG	
AD-CcVPS29-R	cagctcgagctcgatggatccCTATGATACACCAGCGCCG	
CcVPS35-Myc-CF	agggaacaaaagctgggtaccCAACGATCGCTTGTCGCCC	
CcVPS35-Myc-CR	gccgaattcgatatcaagcttTTACAGGTCCTCCTCTGAGATCAGCTTCTGCTCTTTTGGCGCTGTCACGACACGA	
CcVPS35QF	ATGGCGTCGCCGCCCCC	For-qRT-PCR
CcVPS35QR	CTATTTTGGCGCTGTCACG	
CcVPS29QF	ATGGCTTCCCGCCTCGTG	
CcVPS29QR	CTATGATACACCAGCGCCG	
CcVPS26QF	ATGGCGTCGCCGCCCCC	
CcVPS26QR	CTATTTTGGCGCTGTCACG	
Actin-F	GCGACCGACAATGGAAGCT	
Actin-R	CGATGCCCGCCAGCAAAGC	
qΔCcVps35OF	ACAAGCTATGGGTGCGACTG	
qΔCcVps35OR	CCACAAGCTGGCTCAGACG	

qΔCcVps29OF	AGGGCGACTTCGATGTCGA	
qΔCcVps29OR	CCCGTGCGTGAAGCCTAT	
qΔCcVps26OF	TCAACGTCAAGCTGCGCTA	
qΔCcVps26OR	TGGAGCTGTTGGTCTCTGGA	
qΔCcSnc1OF	CATTGTCCCCTTCACATGCAC	
qΔCcSnc1OR	GCGCTCAGAGACCTTGTTG	

Table S4. The plasmids used in this study.

Clone	Description
pCcVps35-GFP	For expression of CcVps35: GFP, cloned in KpnI-HindIII sites of pKNT-GFP. Ampicillin and Neomycin resistance
pCcVps29-GFP	For expression of CcVps29: GFP, cloned in KpnI-HindIII sites of pKNT-GFP. Ampicillin and Neomycin resistance
pCcVps26-GFP	For expression of CcVps26: GFP, cloned in KpnI-HindIII sites of pKNT-GFP. Ampicillin and Neomycin resistance
pCcVps35-mCheery	For expression of CcVps35: mCheery, cloned in KpnI-HindIII sites of pKNT- mCheery. Ampicillin and Neomycin resistance.
pCcKex2-mCheery	For expression of CcKex2: mCheery, cloned in KpnI-HindIII sites of pKNT- mCheery. Ampicillin and Neomycin resistance.
pCcSpa2-mCheery	For expression of CcSpa2: mCheery, cloned in KpnI-HindIII sites of pKNT- mCheery. Ampicillin and Neomycin resistance.
pmCherry-CcRab52	For expression of mCherry: CcRab52, cloned in KpnI-BamHIII sites of pKNT. Ampicillin and Neomycin resistance
pCcSnc1-GFP	For expression of GFP: CcSnc1, cloned in KpnI-BamHIII sites of pKNT. Ampicillin and Neomycin resistance
pBD-CcVps29	CcVps29 was cloned in pDHB1. Kanamycin resistance.
pBD-CcVps26	CcVps26 was cloned in pDHB1. Kanamycin resistance.
pAD-CcVps35	CcVps35 was cloned in pPR3-N. Ampicillin resistance
pAD-CcVps29	CcVps29 was cloned in pPR3-N. Ampicillin resistance

Table S5. Summary of Illumina sequencing and transcriptome assemblies for RNA-Seq libraries.

Sample	Raw reads	Clean reads	Clean bases(G)	Q20(%)	Q30(%)	Error Rate(%)
C0h_1	70113680	67771404	10.17	95.52	89.58	0.02
C0h_2	70113680	67871064	10.18	95.39	89.27	0.02
C0h_3	73619364	70898154	10.63	95.24	88.92	0.02
C24h_1	70113680	67433702	10.12	96.07	90.67	0.02
C24h_2	70113680	67545610	10.13	96.16	90.88	0.02
C24h_3	73619364	70231596	10.53	96.04	90.59	0.02
C72h_1	71866522	68789380	10.32	95.97	90.44	0.02
C72h_2	77125048	68934824	10.34	96.13	90.85	0.02
C72h_3	71866522	68863100	10.33	95.98	90.46	0.02

C0h_1、C0h_2、C0h_3：Control group

Table S6. The data of RT-qPCR and RNA-seq at 24 hpi and 72hpi.

Gene ID	$Log_2(2^{-\Delta\Delta Ct})$	log_2FC	$Log_2(2^{-\Delta\Delta Ct})$	log_2FC
BS50DRAFT_176962	-1.980053428	-1.082082183	-2.587537044	-1.03946869
BS50DRAFT_479264	-1.498712956	-2.622352983	-3.49973408	-2.316745819
BS50DRAFT_538366	-5.456583957	-4.281807962	-4.502514534	-5.322565574
BS50DRAFT_569508	-1.480164992	-1.866407819	-0.714842449	-1.340808767
BS50DRAFT_574302	1.298712958	2.21405298	2.994203553	1.781444611
BS50DRAFT_584320	4.932975287	3.629152961	3.798712958	2.158537143
BS50DRAFT_604234	-4.602514534	-5.509893314	-3.456583957	-4.116914029
BS50DRAFT_613819	-4.761530041	-3.647448238	-6.473205481	-5.914612309
BS50DRAFT_629187	-2.758877416	-1.702299303	-1.221335026	-1.529228401
BS50DRAFT_635126	-5.656416094	-6.007775523	-4.656583957	-3.383402724
BS50DRAFT_637300	-3.214842449	-1.287944107	-1.980053428	-0.846690191
BS50DRAFT_674345	12.13890667	14.67110481	11.20520304	12.99678174
BS50DRAFT_675241	7.778990166	5.320786674	2.612450093	3.227620213
BS50DRAFT_573581	11.88689938	12.48947169	11.18612581	12.96470186
BS50DRAFT_652902	14.70402241	13.61597441	14.13990337	13.53363391
